# Effects of coastal saline-alkali soil on rhizosphere microbial community and crop yield of cotton at different growth stages

**DOI:** 10.3389/fmicb.2024.1359698

**Published:** 2024-04-19

**Authors:** Guoyi Feng, Yajie Wu, Chuanzhen Yang, Qian Zhang, Shulin Wang, Ming Dong, Yan Wang, Hong Qi, Lixue Guo

**Affiliations:** ^1^Hebei Branch of National Cotton Improvement Center/Key Laboratory of Cotton Biology and Genetic Breeding in Huanghuaihai Semiarid Area, Ministry of Agriculture and Rural Affairs, Cotton Research Institute Hebei Academy of Agricultural and Forestry Sciences, Shijiazhuang, China; ^2^State Key Laboratory of Cotton Bio-breeding and Integrated Utilization, Institute of Cotton Research of CAAS, Anyang, China

**Keywords:** land salinization, saline-alkali soil, cotton, soil microbial diversity, amplicon sequencing

## Abstract

Soil salinization is a global constraint that significantly hampers agricultural production, with cotton being an important cash crop that is not immune to its detrimental effects. The rhizosphere microbiome plays a critical role in plant health and growth, which assists plants in resisting adverse abiotic stresses including soil salinization. This study explores the impact of soil salinization on cotton, including its effects on growth, yield, soil physical and chemical properties, as well as soil bacterial community structures. The results of β-diversity analysis showed that there were significant differences in bacterial communities in saline-alkali soil at different growth stages of cotton. Besides, the more severity of soil salinization, the more abundance of *Proteobacteria*, *Bacteroidota* enriched in rhizosphere bacterial composition where the abundance of *Acidobacteriota* exhibited the opposite trend. And the co-occurrence network analysis showed that soil salinization affected the complexity of soil bacterial co-occurrence network. These findings provide valuable insights into the mechanisms by which soil salinization affects soil microorganisms in cotton rhizosphere soil and offer guidance for improving soil salinization using beneficial microorganisms.

## 1 Introduction

A saline soil is generally defined as one in which the electrical conductivity of the saturation extract in the root zone exceeds 4 dS m^−1^ (approximately 40 mM NaCl) at 25°C and has an exchangeable sodium of 15% (Shrivastava and Kumar, 2015). Soil salinization is one of the most damaging environmental abiotic stresses, resulting in a substantial decrease in crop yield and quality, due to the accumulation of water-soluble salts in the surface soil layer that exceed normal values and have unfavorable physicochemical properties (Bai et al., 2023). Soil salinization is a global problem, affecting about 23% of the world’s arable land (Tang et al., 2023). In addition, salinized areas are growing at a rate of 10% per year due to low precipitation, large surface evaporation, weathering of native rocks, irrigation with saline water, and poor cultural practices. It is expected that more than 50% of arable land will be salinized by 2050 (Jamil and Ashraf, 2011). The salinization of arable land is also a common environmental disaster in coastal areas of China (Cui et al., 2021). Soil salinization in coastal areas is the result of multiple factors, including the transport of rivers, the toppling of seawater, the ocean currents, tides, and waves (Yang et al., 2021). A large acreage of quality land is coming under salinity every year. This poses serious limitations to crop productivity and limits sustainable land use. Any attempt to reduce the effects of salinity in plant systems may improve crop growth at high salinity. The amelioration of coastal saline soils is essential to sustainable agriculture in coastal zones. This could be an option to fill the gap in crop demand (Gupta et al., 2023).

Soil salinity is a prominent abiotic stressor that significantly impedes crop growth, with its impact intensifying over time (Chaves et al., 2009). Soil salinization imposes substantial limitations on achieving the full yield potential of crop cultivars. The escalating levels of soil salinization pose a grave threat to the overall crop production system, resulting in a sharp decline in both the quality and quantity of agricultural produce. The expansion of saline soil is expedited by intensive agricultural practices. Furthermore, salinity stress in plants adversely affects nutrient uptake, osmotic balance, membrane integrity, and overall growth, thereby disrupting the dynamics of the entire crop (Zhang et al., 2022). It also causes generation of excessive reactive oxygen species, which besides acting as signaling molecule, it can harm plant function and reduce productivity at higher concentrations (Czarnocka and Karpinski, 2018). A large acreage of quality land is coming under salinity every year. This poses serious limitations to crop productivity and limits sustainable land use.

Plant rhizosphere microhabitat is the most active microhabitat in soil, and it is also the main area for plants to obtain nutrients. In this microdomain, plant-microbial-soil-environment interactions jointly maintain the balance of the rhizosphere microecosystem and affect crop production. Plant-associated microbes play an important role in host nutrient utilization, stress tolerance, plant health, and adaptation (Yang et al., 2022; Pang et al., 2023). Soil microorganisms are not only the drivers of soil organic matter turnover and nutrient cycling, but also an important part of soil organic matter. Therefore, any effect of salt accumulation on microorganisms will affect the turnover process of soil organic matter (Setia et al., 2012). On the one hand, microorganisms reduce soil organic matter content through mineralization; on the other hand, microbial dead bodies in the turnover process of microorganisms account for 10–80% of soil organic matter content (Liang et al., 2019; Wang et al., 2021). Therefore, soil microorganisms act as “carbon pumps” in the turnover process of soil organic matter. It has been reported that microorganisms have a close relationship with plants in nutrient cycling and mitigating biotic and abiotic stresses (Meena et al., 2017). Microorganisms have the ability to maintain plant growth under salt-stress conditions, such as improving nutrient uptake, osmotic balance, ion balance, membrane stability, and overall growth (Sahu et al., 2021). Microorganisms that can mitigate the deleterious effects of soil salinization on plant growth and productivity are being exploited for sustainable agriculture, exploring that rhizosphere microorganisms can ameliorate the adverse effects of salt stress on different physiological parameters (Gupta et al., 2023).

In the agriculture sector, cotton is an important cash crop, which has strong adaptability to saline-alkali soil and is a pioneer crop for saline-alkali soil improvement (Guo et al., 2015). Given the increasing detrimental impacts of saline-alkali soil, it is important to characterize the different microbial systems of major crops such as cotton, so that the base of effective climate-adaptive cropping strategies can be expanded to prepare Chinese agriculture for the future. Therefore, it is necessary to evaluate the potential of cotton rhizosphere microorganisms in alleviating salt stress and excavate beneficial rhizosphere microorganisms.

## 2 Materials and methods

### 2.1 Experimental design

The tested soil is coastal salinized tidal soil, characterized by low nutrient content, inadequate water and fertilizer retention capabilities, and susceptibility to drought stress. In the field experimental station located in Haixing County, Cangzhou city, Hebei province, State-owned Haixing farm, cotton rhizosphere soil samples were collected from different saline-alkali fields and different growth periods. The coordinates provided represent the locations of the fields with slight saline-alkali soil (38°21’N, 117°31′E), moderate saline-alkali soil (38°03’N, 117°39′E), and heavy saline-alkali soil (38°12’N, 117°34′E). The saline-alkali soil was classified according to the salt content of 0–20 cm soil layer: mild saline-alkali soil (0.1–0.25%), moderate saline-alkali soil (0.25–0.45%), and severe saline-alkali soil (0.45–0.8%). Jimian 228 is utilized as the experimental material, and cotton from different experimental groups is sown and managed under the same conditions during the same period. The sampling time was April 15, 2022 at the seedling stage, July 7, 2022 at the flowering stage, and October 12, 2022 at the boll opening stage. There were nine groups of samples in the experiment: slight saline-alkali soil sample at the seedling stage (SS), moderate saline-alkali soil sample at the seedling stage (MS), heavy saline-alkali soil sample at the seedling stage (HS), slight saline-alkali soil sample at the flowering stage (SF), moderate saline-alkali soil sample at the flowering stage (MF), and heavy saline-alkali soil sample at the flowering stage (HF). Soil samples from slight saline-alkali soil during boll opening stage (SB) Soil samples from moderate saline-alkali soil during boll opening stage (MB) soil samples from heavy saline-alkali soil during boll opening stage (HB). Three samples were taken from each group.

### 2.2 Soil sample collection and analysis

Immediately after the roots were brought back to the laboratory in a fresh-keeping container, rhizosphere soil samples were collected to remove impurities and stones. For each sample, 100 g of screened soil was selected for aggregate classification. The roots were washed with sterile water, the mixture was centrifuged, and the precipitated soil sample was stored at −80°C for bacterial community analysis (Tao et al., 2020). The residual portions of soil samples were further air-dried for physicochemical property determination.

The content of organic carbon (SOC), total nitrogen (TN), total phosphorus (TP), total potassium (TK), alkali-hydrolyzable nitrogen (AHN), available phosphorus (AP) and available potassium (AK) in different soil samples was measured according to the methods described by previous researchers (Zhang et al., 2021). The wet sieving method was employed to separate soil water-stable aggregates using an agglomerate analyzer (Olsen and Sommers, 1982; Elliott, 1986).

### 2.3 DNA extraction and amplicon sequencing

Total genomic DNA was extracted from 27 samples using the TGuide S96 Magnetic Soil DNA Kit (Tiangen Biotech (Beijing) Co., Ltd.) according to manufacturer’s instructions. DNA was detected on a 1% agarose gel, and the concentration was determined using a spectrophotometer NanoDrop 2000 (NanoDrop Technologies, United States). The hypervariable region V3–V4 of the bacterial 16S rRNA gene were amplified with primer pairs 338F: 5′-ACTCCTACGGGAGGCAGCA-3′ and 806R: 5′-GGACTACHVGGGTWTCTAAT-3′. Both the forward and reverse 16S primers were tailed with sample-specific Illumina index sequences to allow for deep sequencing. The PCR was performed in a total reaction volume of 10 μL: DNA template 5–50 ng, forward primer (10 μM) 0.3 μL, reverse primer (10 μM) 0.3 μL, KOD FX Neo Buffer 5 μL, dNTP (2 mM each) 2 μL, KOD FX Neo 0.2 μL, and finally ddH2O up to 20 μL. After with initial denaturation at 95°C for 5 min, followed by 20 cycles of denaturation at 95°C for 30 s, annealing at 50°C for 30 s, and extension at 72°C for 40 s, and a final step at 72°C for 7 min. The amplified products were purified with Omega DNA purification kit (Omega Inc., Norcross, GA, United States) and quantified using Qsep-400 (BiOptic, Inc., New Taipei City, Taiwan, ROC). The amplicon library was paired-end sequenced (2 × 250) on an Illumina novaseq6000 (Beijing Biomarker Technologies Co., Ltd., Beijing, China).

### 2.4 Bioinformatic analysis

In order to ensure the quality and reliability of the sequencing data, a series of quality control steps were performed. The raw reads obtained from sequencing were subjected to quality filtering using Trimmomatic (version 0.33) software, which removed low-quality bases and reads based on predefined thresholds (Bolger et al., 2014). Cutadapt (version 1.9.1) software was used to identify and remove primer sequences from the reads, resulting in clean reads devoid of any primer residues (MARTIN 2011). PE reads obtained from previous steps were assembled by USEARCH (version 10) and followed by chimera removal using UCHIME (version 8.1) (Edgar et al., 2011; Edgar, 2013).

Clean reads then were conducted on feature classification to output an ASVs (amplicon sequence variants) by dada2 (Callahan et al., 2016), and the ASVs conuts less than 2 in all samples were filtered. Taxonomy annotation of the OTUs was performed based on the Naive Bayes classifier in QIIME2 (Bolyen et al., 2019) using the SILVA database (release 138.1) (Quast et al., 2013) with a confidence threshold of 70%. The alpha diversity measures were calculated and displayed using the QIIME2 software for diversity analysis and the R software for visualization. Beta diversity was determined to evaluate the degree of similarity of microbial communities from different samples using QIIME. Principal coordinate analysis (PCoA) and nonmetric multidimensional scaling (NMDS) were used to analyze the beta diversity. PCoA (Gower, 1966) was performed to analyze the species diversity differences among multiple samples. The analysis utilized four distance matrices obtained from Beta diversity analysis, and the PCoA results were visualized using R language tools. NMDS (Looft et al., 2012) are used to reduce the research objects (samples or variables) in multidimensional space to low-dimensional space for localization, analysis and classification, while preserving the original relationship between objects. Furthermore, we employed Linear Discriminant Analysis (LDA) effect size (LEfSe) (Segata et al., 2011) to test the significant taxonomic difference among group. A logarithmic LDA score of 4.0 was set as the threshold for discriminative features. To investigate the differences in microbiome composition among different factors, a redundancy analysis (RDA) was conducted using the “vegan” package in R. Gtree Extra was used for visualization in the form of circular evolutionary tree and bar chart to obtain the evolutionary relationship between species and the relative abundance ratio of species among different soil samples.

### 2.5 Statistical analysis

SPSS Statistics was used to analyze the data, calculate the standard deviation of each experimental group, and perform the one-way ANOVA. The significant differences were determined based on *p* < 0.05 using the least significance difference (LSD) analysis. GraphPad Prism was used to generate the graphs with statistical analysis.

## 3 Results

### 3.1 Soil characteristics and cotton yield in saline-alkali soil

Soil mechanical stability aggregate can resist mechanical damage and is one of the indicators to evaluate the quality of soil structure. At the seedling stage, large aggregates, small aggregates and the total number of aggregates in SS were significantly higher than those in HS. Both the total number of small aggregates and aggregates in SB were significantly higher than those in MB and HB at the boll opening stage (Table 1). Table 2 presents the chemical properties of different saline-alkali soils. The results indicate that the SOC, TN, TP, TK, AHN, AP and AK content in slight saline-alkali soil are significantly higher than those in moderate saline-alkali soil and heavily saline-alkali soil. However, there were no significant differences observed in these indicators between moderate saline-alkali soil and heavily saline-alkali soil. Severe soil salinization has a significant impact on cotton yield. In heavily saline-alkali soil, the boll weight, number of plants harvested per hectare, number of bolls per plant, and yield per hectare are significantly lower compared to slight saline-alkali soil and moderate saline-alkali soil. Additionally, moderate saline-alkali soil also exhibits a significantly lower cotton yield compared to slight saline-alkali soil (Table 3).

**Table 1:** Mechanical stable aggregate ratio of different soil samples.

Factor	5 mm	2 mm	Macro aggregate	1 mm	0.5 mm	0.25 mm	Small aggregate	Total
	Proportion (%)							
SS	1.010 ± 0.44a	0.235 ± 0.10ab	1.244 ± 0.35a	0.365 ± 0.15bc	0.511 ± 0.11b	0.816 ± 0.11ab	1.692 ± 0.64b	2.937 ± 0.22a
MS	0 b	0.149 ± 0.03 b	0.149 ± 0.03 b	0.202 ± 0.05 cd	0.435 ± 0.07 b	0.769 ± 0.08 ab	1.406 ± 0.17 bcd	1.554 ± 0.07 bc
HS	0.206 ± 0.11 b	0.158 ± 0.05 b	0.364 ± 0.08 b	0.152 ± 0.04 cd	0.205 ± 0.06 c	0.433 ± 0.08 cd	0.789 ± 0.25 de	1.153 ± 0.071 cd
SF	0.136 ± 0.06 b	0.093 ± 0.02 b	0.228 ± 0.06 b	0.244 ± 0.04 bcd	0.362 ± 0.03 bc	0.391 ± 0.02 cd	0.997 ± 0.15 cde	1.225 ± 0.06 bcd
MF	0.054 ± 0.05 b	0.102 ± 0.05 b	0.156 ± 0.05 b	0.165 ± 0.01 cd	0.322 ± 0.02 bc	0.312 ± 0.04 cd	0.798 ± 0.06 de	0.954 ± 0.019 cd
HF	0.016 ± 0.02 b	0.062 ± 0.02 b	0.077 ± 0.02 b	0.123 ± 0.02 d	0.215 ± 0.01 c	0.224 ± 0.01 d	0.562 ± 0.05 e	0.639 ± 0.05 d
SB	0.111 ± 0.11 b	0.470 ± 0.15 a	0.580 ± 0.26 b	0.876 ± 0.07 a	1.096 ± 0.07 a	0.945 ± 0.21 bc	2.917 ± 0.56 a	3.497 ± 0.58 a
MB	0.005 ± 0.01 b	0.466 ± 0.08 a	0.470 ± 0.07 b	0.469 ± 0.04 b	0.427 ± 0.07 b	0.559 ± 0.05 a	1.455 ± 0.05 bc	1.926 ± 0.05 b
HB	0.079 ± 0.01 b	0.202 ± 0.02 b	0.28 ± 0.02 b	0.261 ± 0.05 bcd	0.388 ± 0.02 bc	0.401 ± 0.03 cd	1.050 ± 0.12 cde	1.330 ± 0.09 bcd

Same letters in a column in the table show no significant difference at 0.05 level.

**Table 2:** The chemical properties of different soils.

Factor	SOC	TN	TP	TK	AHN	AP	AK
SSA	7.34 ± 0.143 a	0.521 ± 0.036 a	0.741 ± 0.0144 a	20.596 ± 0.194 a	26.425 ± 2.134 a	10.347 ± 0.678 a	161.167 ± 2.055 a
MSA	3.121 ± 0.223 b	0.303 ± 0.096 b	0.567 ± 0.0107 b	18.666 ± 0.261 b	11.492 ± 0.644 b	1.492 ± 0.047 b	46.833 ± 4.7842 b
HSA	3.118 ± 0.248 b	0.243 ± 0.008 b	0.568 ± 0.01 b	19.398 ± 0.183 b	14.467 ± 1.091 b	1.742 ± 0.238 b	48.167 ± 2.494 b

SSA, slight saline-alkali soil; MSA, moderate saline-alkali soil; HSA, heavily saline-alkali soil; SOC, soil organic carbon; TN, total nitrogen; TP, total phosphorus; TK, total potassium; AHN, alkali-hydrolyzable nitrogen; AP, available phosphorus; AK, available potassium. Same letters in a column in the table show no significant difference at 0.05 level.

**Table 3:** Effect of different saline-alkali soil on cotton yield.

	Single bell weight (g)	Number of harvested plants (10,000 plants/ha)	Boll number per plant	Yield (kg/ha)
SSA	5.9225 ± 0.097 a	4.246 ± 0.097 a	15.071 ± 0.3511 a	3218.2328 ± 10.6421 a
MSA	5.6267 ± 0.0985 b	3.7525 ± 0.0985 b	13.633 ± 0.0471 b	2447.8567 ± 83.99 b
HSA	4.9472 ± 0.0988 c	3.1605 ± 0.0988 c	11.2282 ± 0.2702 c	1491.66 ± 56.8541 c

SSA, slight saline-alkali soil; MSA, moderate saline-alkali soil; HSA, heavily saline-alkali soil. Same letters in a column in the table show no significant difference at 0.05 level.

### 3.2 Alpha diversity

The number of raw reads for the 24 samples ranged from 42,938 to 115,762, with an average of 75,580. The number of original sequences after quality control, denoising and removal of chimeras ranged from 36,066 to 90,870, with an average of 63,242. The sequences clustered into ASVs with 99% similarity, were identified as 39,503 ASVs in total. The Rarefaction curves randomly selected a certain number of sequences from the sample, counted the number of species represented by these sequences, and constructed a curve based on the number of sequences and the number of species to verify whether the amount of sequencing data was sufficient to reflect the species diversity in the sample. The results showed that the dilution curves of the soil samples were basically flat, indicating that the sequencing was saturated and the sample sequences were sufficient for data analysis (Supplementary Figure S1).

**Figure 1:**
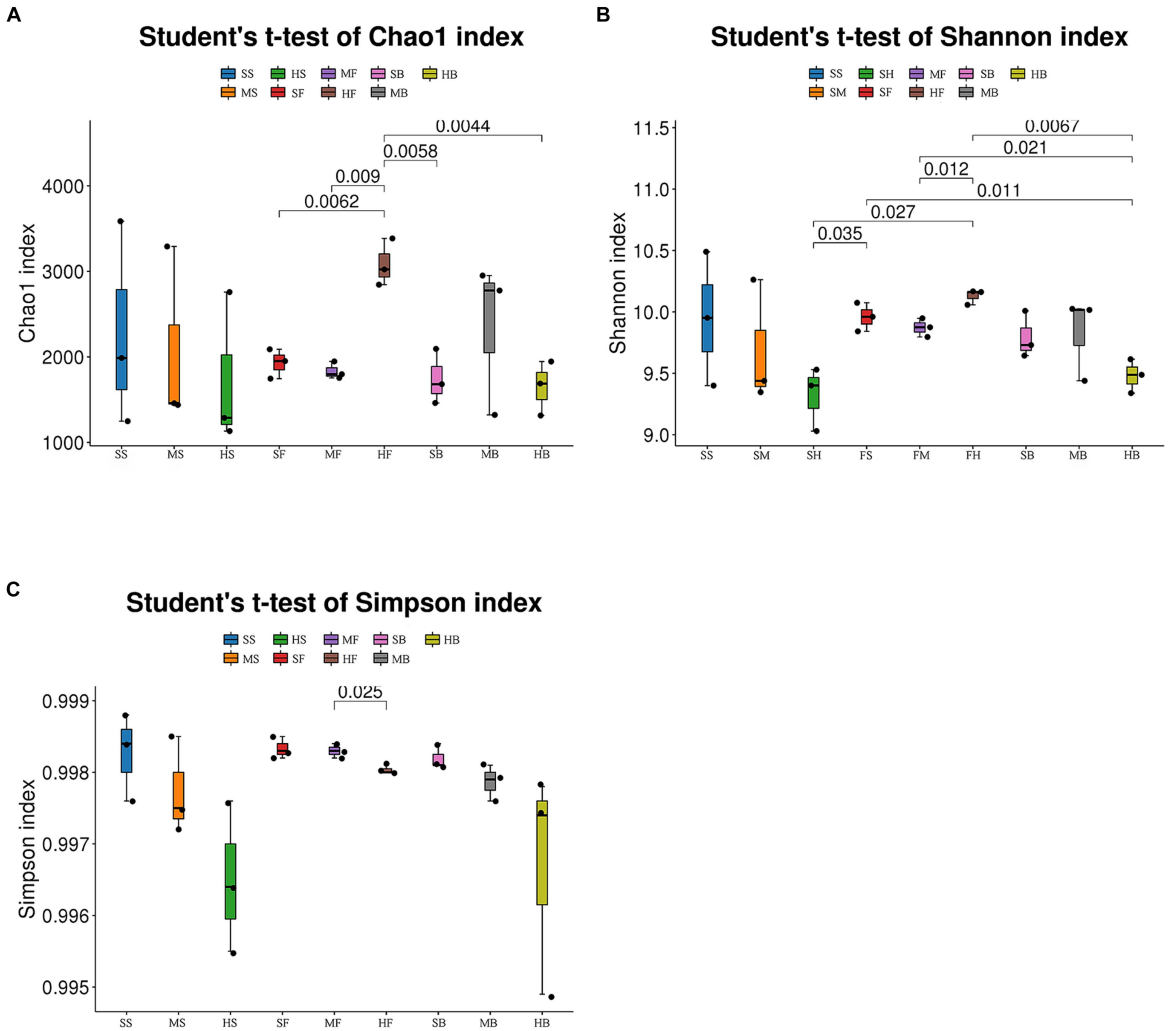
Chao1 index **(A)**, Simpson index **(B)**, and Shannon index **(C)** of bacterial communities in soil samples from cotton fields in different saline-alkali soils. In the figure, SS: mild saline-alkali land at seedling stage, MS: moderate saline-alkali land at seedling stage, HS: severe saline-alkali land at seedling stage, SF: mild saline-alkali land at flower and boll stage, MF: moderate saline-alkali land at flower and boll stage, HF: severe saline-alkali land at flower and boll stage, SB: mild saline-alkali land at boll opening stage, MB: moderate saline-alkali land at boll opening stage, HB: severe saline-alkali land at boll opening stage. Numbers in the figures indicate *p*-values across treatments (Student’s *t*-test).

QIIME2 2020.6 software was used to evaluate the Alpha diversity index of the samples to reflect the species richness and diversity of a single sample. Alpha diversity analysis showed that Chao1 index, Shannon index and Simpson index of cotton rhizosphere bacterial communities in heavy saline soil tended to be lower than that in light saline soil at seedling stage and boll opening stage (without significant difference, student’s *t*-test). At the boll opening stage, the Simpson index of cotton rhizosphere soil in heavy saline-alkali soil was significantly lower than that in light saline-alkali soil (Figure 1). These results indicating that the degree of soil salinization is related to the diversity of soil bacterial communities.

### 3.3 Beta diversity

**Figure 2:**
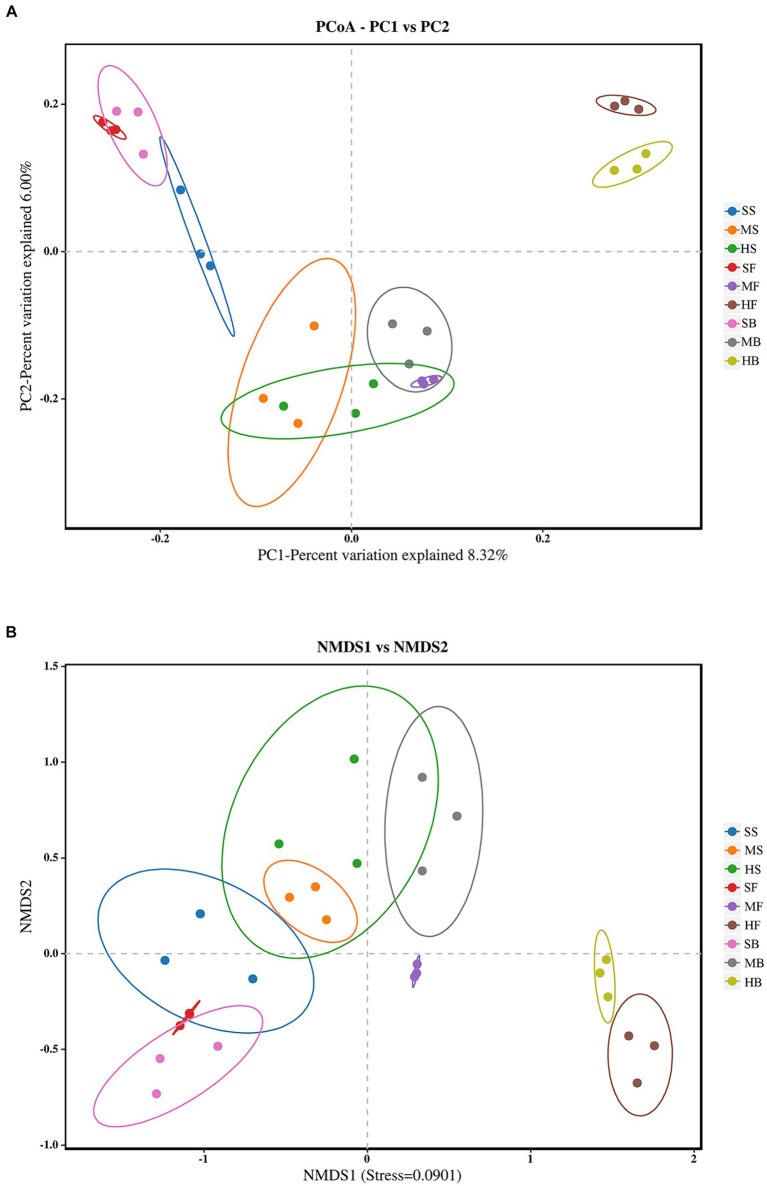
**(A)** Principal coordinate analysis (PCoA) of bacterial community structure in different saline-alkali soil samples based on binary_jaccard algorithm. **(B)** Non-metric-multi-dimensional Scaling (NMDS) analysis of different saline-alkali soil samples based on binary_jaccard algorithm. The oval circle indicates that it is a 95% confidence ellipse.

Beta diversity based on Bray–Curtis analysis was carried out to compare the viration of bacterial community structures among different samples. PCoA was used to analyze the effects of different soil samples on bacterial community structure (Figure 2A). Samples of HF and HB was separated with the other seven treatments by PC1 (22.59%); SS, SF, SB samples were gathered at the top left corner of the graph, by PC2 (12.67%). MS, HS, MB and MF showed similar bacterial community structures, as they were located in the bottom part of PC2.

NMDS based on binary_jaccard distance can be employed to reveal the variations in soil microbial community structure among different sample treatments. The results were similar to the PCoA analysis, with significant changes in bacterial communities in different soil samples. HF and HB in the heavily saline soil were significantly separated from the rest of the soil samples along the NMDS1 axis, and the three treatments SS, SF, and SB in the lightly saline soil were close to each other, indicating that the bacterial community structure was relatively similar (Figure 2B). This suggests that the saline soil has effects on the microbial community structure.

Based on the information provided, Table 4 indicates that the degree of soil salinization and the growth period have a significant influence on the soil microenvironment. The results of the PERMANOVA analysis using Bray-curtis distance indicate significant differences in soil bacterial communities among slight, moderate, and heavy saline-alkali soils (*R*^2^ = 0.151, *p* = 0.003). Additionally, there are notable distinctions in soil bacterial communities during different growth stages, including seedling, flowering, and boll opening stages (*R*^2^ = 0.226, *p* = 0.001). These results suggest that both the degree of soil salinization and the growth stage significantly impact the composition of soil bacterial communities.

**Table 4:** PERMANOVA analysis of different grouped cotton rhizosphere soil microbial community structures.

Parameter		F. Model	Variation (*R*^2^)	Pr (>*F*)
Bacteria	Degree of soil salinity	2.125971631	0.150500913	0.003
	Period of duration	3.507699112	0.226190816	0.001

### 3.4 Bacterial community composition

**Figure 3:**
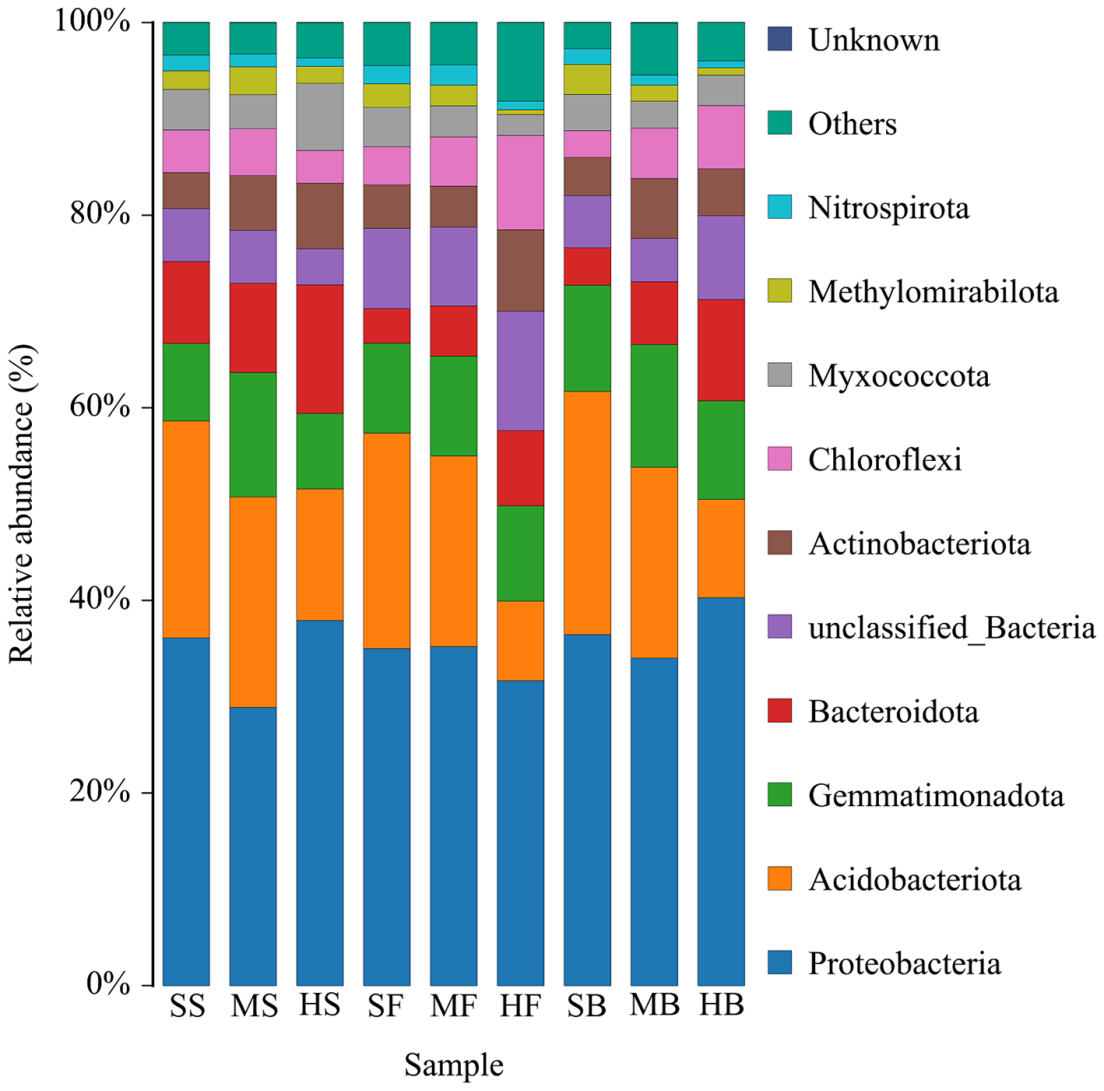
Relative abundance of the top 12 bacterial phyla in cotton rhizosphere soil samples from different saline-alkali soil.

Furthermore, we conducted an analysis of the composition of soil bacterial communities to examine the impact of soil salinity differences on species diversity. In total, we identified 42 bacterial phyla across all samples. Among them, *Proteobacteria*, *Actinobacteria*, *Gemmatimonadota*, *Bacteroidota*, *Actinobacteriota*, and *Chloroflexi* were the dominating bacterial phyla with 80% relative abundance totally. Moreover, *Proteobacteria* was the most dominant bacterial phylum accounting for 30% relative abundance (Figure 3).

### 3.5 Sample community distribution map

**Figure 4:**
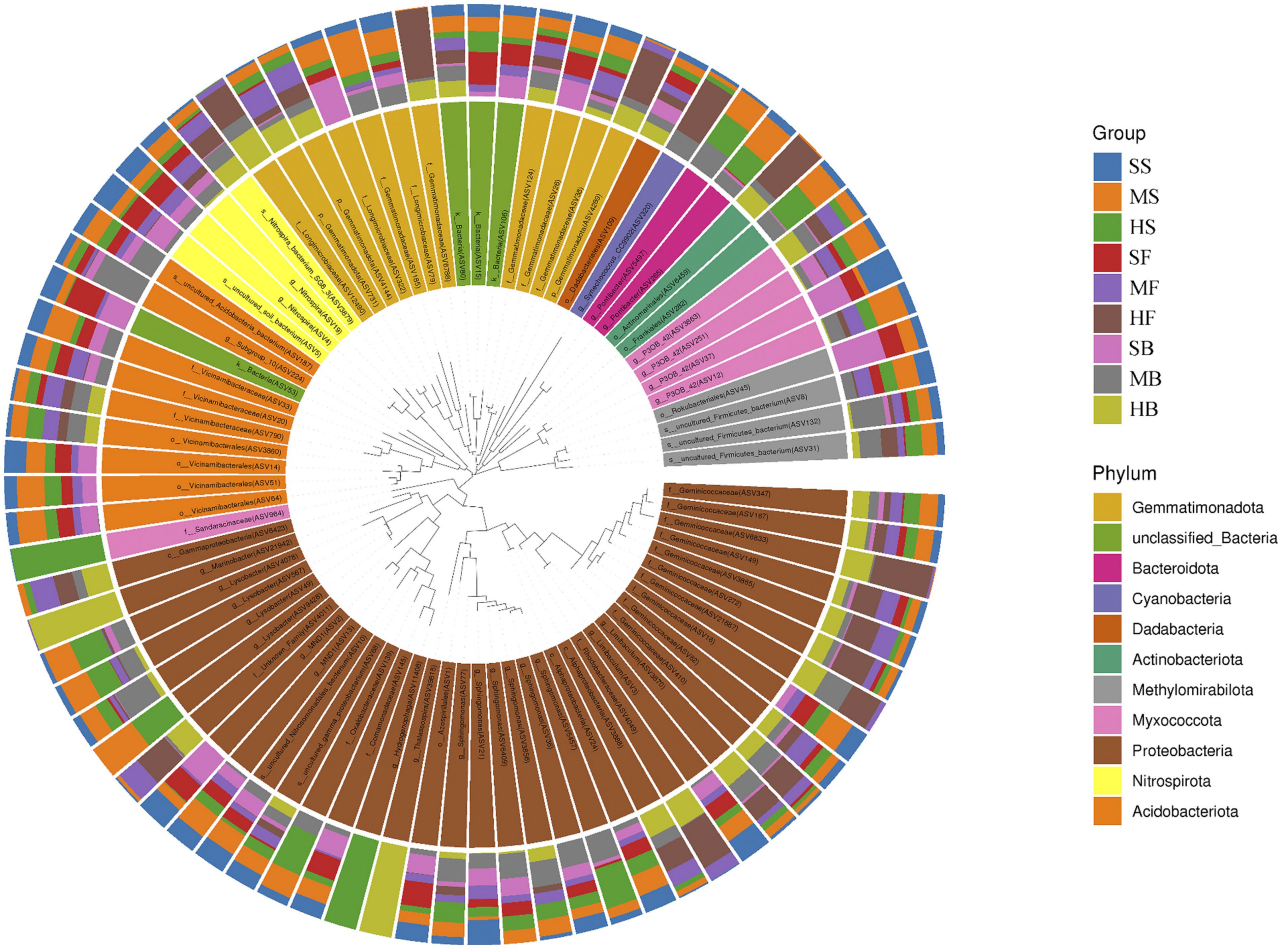
Sample community distribution map of the species evolutionary tree.

The characteristic sequences with the relative abundance ratio Top80 of ASV at the taxonomic level were selected to obtain the evolutionary relationship between species and the relative abundance ratio of species among different soil samples (Figure 4). According to the figure, ASV11408 (*g_Hydrogenophaga*) was present only in the HS group, ASV39815 (*g_Thalassospira*) was present only in the GH group, and ASV49 (*g_Lysobacter*) was present only in the HS and MS groups, ASV21942 (*g_Marinobacter*) was only present in the GH and FM groups. In additions, ASV132 (*g_Firmicutes bacterium*), ASV251 (g_P30B_42), ASV109 (*o_Dadabacteriales*), ASV124 (*f_Gemmatimonadaceae*), ASV731 (*f_Gemmatimonadota*), ASV3879 (*s_Nitrospira_bacterium_SG8_3*), ASV19 (*g__Nitrospira*), ASV49r (*g_Lysobacte*), ASV88 (*s_uncultured_gamma_proteobacterium*), ASV410 (*f_Geminicoccaceae*), ASV62 (*f_Geminicoccaceae*), ASV272 (*f_Geminicoccaceae*), ASV149 (*f_Geminicoccaceae*), ASV167 (*f_Geminicoccaceae*), ASV347 (*f_Geminicoccaceae*) was present in all nine sets of soil samples.

### 3.6 Line discriminant analysis

**Figure 5:**
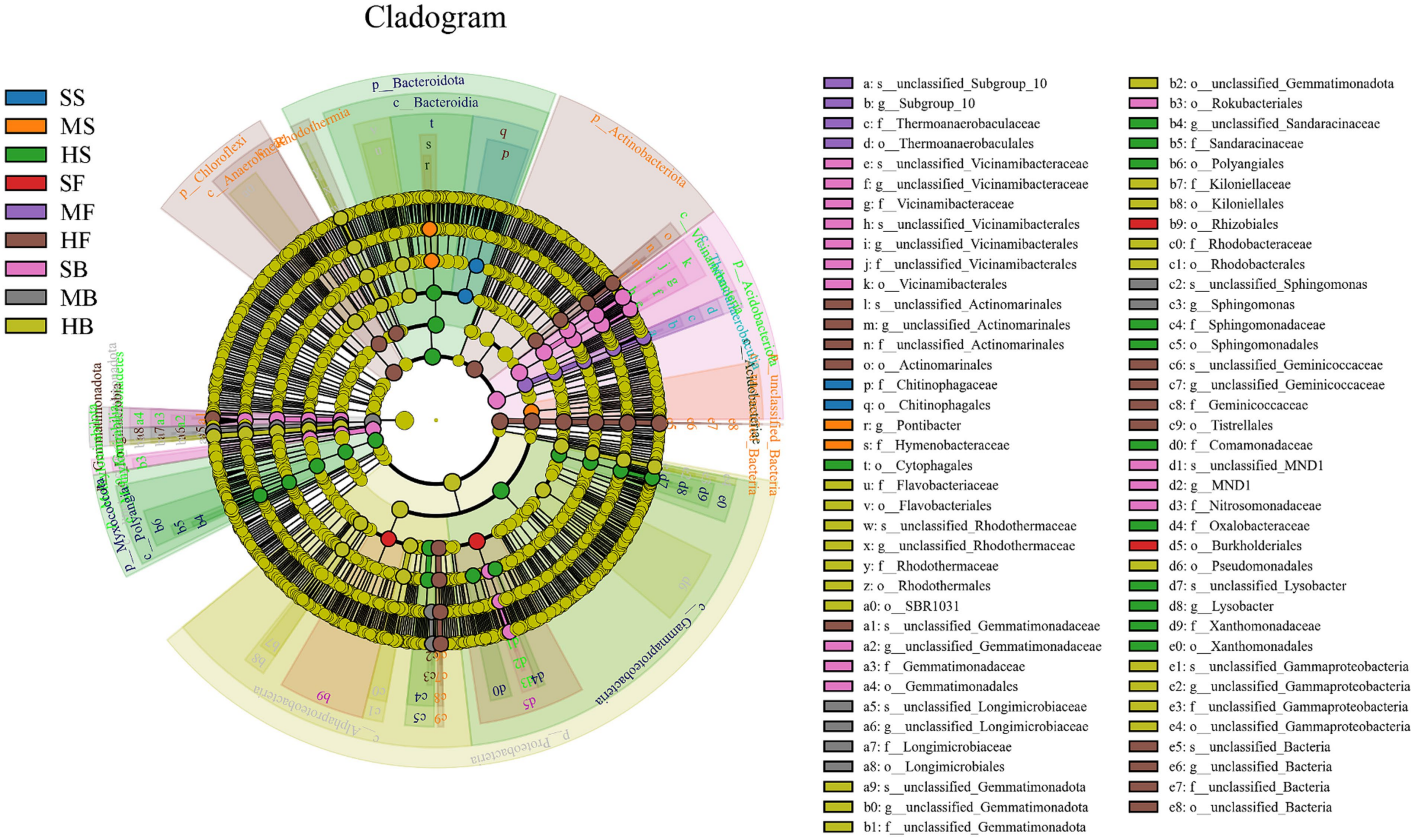
Evolutionary branching diagram of LEfSe analysis.

Line Discriminant Analysis (LDA) Effect Size (LEfSe) method was used to analyze the bacterial taxa with significant differences in abundance in different soil samples. A total of 75 taxa showed significant differences in relative abundance across soils (Figure 5 and Supplementary Figure S2). *o_Chitinophagales* was significantly enriched in cotton rhizosphere soil at seedling stage in light saline-alkali soil. *f_Hymenobacteraceae* was significantly enriched in cotton rhizosphere soil at seedling stage in moderate saline-alkali soil. *o_Cytophagales*, o_Polyangiales, in cotton rhizosphere soil at seedling stage in heavy saline-alkali soil: *o_Sphingomonadales*, *f_Comamonadaceae*, *f_Oxalobacteraceac*, and *o_Xanthomonadales* were significantly enriched. *o_Rhizobiales* and *o_Burkholderiales* were significantly enriched in cotton rhizosphere soil at flowering stage in light saline-alkali soil. *o_Thermoanacrobaculales* was significantly enriched in cotton rhizosphere soil at flowering stage in moderate saline-alkali soil. *o_Actinomarinales* was significantly enriched in cotton rhizosphere soil at flowering stage in heavily saline-alkali soil. *o_Vicinamibacterales*, *o_Gemmatimonadales*, *o_Gemmatimonadales* and *o_Gemmatimonadales* weresignificantly enriched in cotton rhizosphere soil at the boll filling stage in light saline-alkali soil. *o_Longimicrobiales* was significantly enriched in cotton rhizosphere soil at the boll filling stage in moderate saline-alkali soil. *o_SBR1031*, *o_Kiloniellales*, *o_Rhodobacterales*, and *o_Pseudomonadales* were significantly enriched in cotton rhizosphere soil at the boll opening stage in heavily saline-alkali soil.

### 3.7 Co-occurrence network

**Figure 6:**
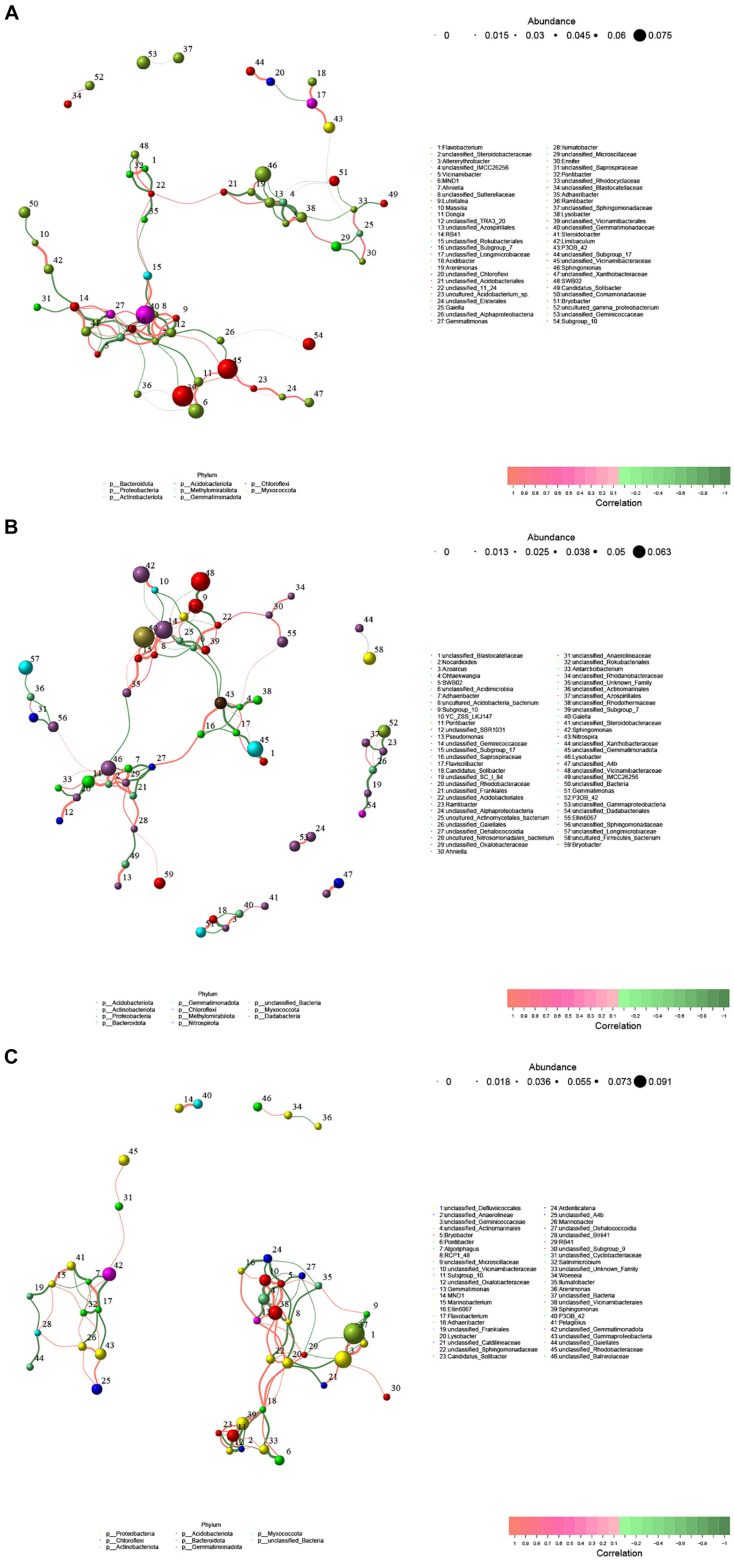
Co-occurrence networks of bacterial communities in soil samples from different salinized cotton fields. **(A)** Lightly saline-alkali land, **(B)** heavily saline-alkali land, **(C)** heavily saline-alkali land.

In order to compare the soil type or developmental stage of soil bacteria colony size and the influence of the interaction of the bacteria, this study screened Spearman rank correlation analysis and correlation is greater than 0.1 and the *p* value is less than 0.05 correlation data to construct the network (Figure 6 and Supplementary Table S1). The results of genus level co-occurrence analysis in the rhizosphere soil of cotton field in slight saline-alkali soil showed that the number of nodes was 54, the average degree of nodes was 3.703704, the average path length was 5.3219, the network diameter was 33.3, the graph density was 0.06988, the clustering coefficient was 0.47561, and the betweenness centralization was 0.40207. The degree centralization was 0.118798, and the modularity was 0.59645. The co-occurrence analysis in the moderate saline-alkali soil showed that the number of nodes was 59, the average degree of nodes was 3.389831, the average path length was 3.72553, the network diameter was 24.81818, the graph density was 0.058445, the clustering coefficient was 0.45205, and the betweenness centralization was 0.15331. The degree centralization was 0.11397, and the modularity was 0.6904. The co-occurrence analysis in the heavily saline-alkali soil showed that the number of nodes was 46, the average degree of nodes was 4.347826, the average path length was 2.39013, the network diameter was 11.7889, the graph density was 0.096618, the clustering coefficient was 0.5077, and the betweenness centralization was 0.10011. The degree centralization was 0.12560, and the modularity was 0.63685. Compared with the slight saline-alkali soil, the heavy saline-alkali soil reduced the total nodes, average path length, graph diameter, betweenness centralization of the network and increased average degree, graph density, clustering coefficient, degree centralization, modularity.

## 4 Discussion

Soil salinity is a widespread global issue that impacts approximately 20% of irrigated land (Shrivastava and Kumar, 2015). It poses a significant challenge to agriculture as it leads to a substantial reduction in crop yields. The presence of excessive salts in the soil interferes with plant growth and development, affecting nutrient uptake, water absorption, and overall plant health. This reduction in crop productivity has detrimental effects on food security and agricultural sustainability. Addressing soil salinity is crucial for maintaining and increasing crop yields to meet the growing demand for food worldwide. In this study, the impact of soil salinization on cotton growth, yield, soil properties, and microbial community structures was examined. The results showed that increasing soil salinity had detrimental effects, reducing soil stability, nutrient availability, and altering the composition of microbial communities. The study also found that cotton yield decreased with higher levels of soil salinization. Additionally, the diversity and structure of microbial communities in cotton rhizosphere soil were affected by soil salinization, with a decline in beneficial microbial groups. These findings provide valuable insights into the mechanisms through which saline-alkali soil affects soil microorganisms in cotton rhizosphere soil and offer guidance for improving saline-alkali soil using beneficial microorganisms.

Soil aggregates are the “cells” of soil and an important habitat for microorganisms. They are composed of soil particles (sand, silt, and clay) cemented with organic or inorganic substances (Six et al., 2000). The higher the proportion of large aggregates, the higher the aggregate stability. Soil aggregate stability affects microbial community structure, regulates oxygen divergence, and regulates water and nutrient transport, which in turn affect the formation and decomposition of soil organic matter (Six et al., 2004). The total amount of soil aggregate decreased with increasing soil salinization at the seedling, flowering and boll and flocculation stages, indicating that soil stability decreased with increasing salinity (Table 1). The contents of soil organic carbon, organic matter, AHN, AP, and AK are considered to be one of the important factors affecting soil bacterial community structure. These factors can affect soil physical and chemical properties and nutritional status, and then affect the survival and activities of soil microorganisms (Zhao et al., 2014). The contents of AHN, AP and AK in the rhizosphere soil of heavily saline-alkali soil were significantly lower than those of slight saline-alkali soil during the three investigated cotton growth periods (Table 2). Soil salinization is a common abiotic stress that reduces the water-extraction capacity of roots and disrupts plant metabolism, in turn affecting crop growth and yield in agroecosystems on a global scale (Yang et al., 2021). In this study, we evaluated the effects of different levels of saline-alkali soil on cotton yield and found that cotton yield indicators decreased with the increase of soil salinization (Table 3). These results indicate that salinization alters the physical and chemical properties of soil in coastal areas, resulting in decreased soil stability and nutrient availability. In addition, different soil physical and chemical environments may lead to different degrees of promoting, inhibiting, or reversing effects of cotton roots on the same bacterial community. These changes had a significant impact on cotton yield, resulting in decreased productivity.

Alpha diversity is used to measure the richness and diversity of a single soil sample. Chao1 measures the number of species (Eren et al., 2012). Shannon and Simpson indices are used to measure species diversity, which are affected by species richness and Community evenness in the sample community. Under the same species richness, the greater the evenness of each species in the community, the greater the diversity of the community was considered. Higher Shannon index values and higher Simpson index values indicate higher species diversity of the samples (Washington, 1984). The Chao1 index of each component showed that species richness in the heavy saline-alkali soil tended to be lower than that in the light saline-alkali soil. Shannon and Simpson indices showed that species diversity in the heavy saline-alkali soil tended to be lower than that in the light saline-alkali soil (Figure 1). The results of beta diversity analysis showed that there were significant differences in bacterial communities of soil with different degrees of salinization. The SS, FS, and GS of the soil samples from the slight saline-alkali soil were closer. SM, FM, and GM of the moderate saline-alkali soil samples were close to each other. The FH and GH of the heavy saline alkali soil samples were far from the other samples, and the samples between the two types of soil were separated from each other (Figure 2).

Microbial diversity and community structure play crucial roles in soil health. A diverse and balanced microbial community is indicative of a healthy soil ecosystem. Various types of microbes, including bacteria, fungi, protozoa, and viruses, contribute to important soil processes such as nutrient cycling, organic matter decomposition, and disease suppression (Howard and Hanna, 2015; Pereg and Mcmillan, 2015). Plants exert selective pressure on the structural and functional diversity of microbial populations through the root exudation, and in relation to soil properties, plant species, growth stage, and many other stress factors (Gomes et al., 2001). Plants provide abundant ecological niches for various microorganisms, including bacteria, fungi, protozoa, nematodes, and viruses, facilitating the formation of complex symbiotic relationships. These microorganisms interact with plants in the natural environment, participating in plant growth and development, influencing plant functional traits, maintaining species diversity, and promoting community structure stability, thereby establishing a plant–soil feedback effect (Agler et al., 2016). Plant root exudates serve as the basis for such plant–soil feedback, shaping specific microbial communities to meet host growth requirements. Microbial communities and plants, in turn, uphold the stability of structure and function in ecosystems by eliciting symbiotic or immune defense mechanisms (Hacquard et al., 2017). Salinity changes soil bacterial diversity and community structure. The effects of various factors, such as soil structure, texture, pH, water content, salinity, mineral content and organic content, on soil microbial communities have been widely confirmed (Berg and Smalla, 2009; Hill et al., 2019). *Proteobacteria*, *Bacteroidetes*, and *Actinobacteria* were the main bacterial communities in the southeastern coastal saline-alkali land (Li et al., 2018). *Proteobacteria*, one of the largest phyla of soil bacteria, play important roles in biogeochemical cycles, including carbon, nitrogen, sulfur, and iron cycles, and they are well known for their versatility and ability to perform multiple metabolic processes (Spain et al., 2009). *Actinomycetes* are widely distributed in soils and play a crucial role in nutrient cycling and defense mechanisms (Stach and Bull, 2005; Jose and Jha, 2017; Bull and Goodfellow, 2019). They have the ability to survive as endophytes within plant tissues, contributing to nutrient assimilation and promoting plant growth (Vurukonda et al., 2018). Additionally, *Actinomycetes* are known to drive nutrient cycling even in harsh environmental conditions (Bull et al., 1998). The *Acidobacteriota* phylum is highly versatile in adapting to various physiological and ecological conditions. It possesses the capacity to degrade complex carbon-containing compounds. Stressors affecting *Acidobacteriota* may influence the cycling processes of plant-derived organic matter (Singh et al., 2007; Kalam et al., 2020). *Gemmatimonadota* is able to adapt to alkaline and high salinity soils (Malard et al., 2019; Guan et al., 2021), and their abundance is influenced by organic nutrient concentrations in the soil and plays a crucial role in soil ecosystems (Deng et al., 2019; Liu et al., 2021). In this study, *Proteobacteria*, *Actinobacteria*, *Gemmatimonadota*, *Bacteroidota*, *Actinobacteriota*, and *Chloroflexi* were the dominating bacterial phyla in in the cotton rhizosphere soil of saline-alkali soil (Figure 3). Among them, *Proteobacteria* accounted for the highest proportion, which was consistent with the results of previous studies (Tian and Gao, 2014; Qiao et al., 2017). There were differences in rhizosphere bacterial community structure among different developmental stages and soil conditions (Figure 3; Supplementary Table S2). We found that the relative abundance of *Proteobacteria* in the heavily saline-alkali soil was higher than that in the moderately and slight saline-alkali soil at the seedling and boll opening stages of cotton. The relative abundance of *Bacteroidota* increased with increasing soil salinity during these three growth periods. This may be due to cotton increasing the relative abundance of *Proteobacteria* and *Bacteroidetes* by secreting root exudates to promote nutrient cycling in rhizosphere soil, which helps cotton adapt to the harsh environment of saline-alkali soil at different growth stages. The relative abundance of *Actinomycetes* in cotton rhizosphere soil in heavy saline-alkali soil was lower than that in moderate and slight saline-alkali soil, which might be due to the failure of *Actinomycetes* to adapt to high saline-alkali soil environment. Different growth stages can also affect the structure of bacterial communities in the rhizosphere. For example, the relative abundance of *Proteobacteria* in rhizosphere soil at the boll opening stage was higher than that at the seedling and blooming stages in the moderate and heavy saline-alkali soil. In the heavy saline-alkali soil, the relative abundance of *Actinobacteriota* at the seedling stage was higher than that at the boll opening and flowering stages. These results suggest that the relative abundance of microorganisms in the cotton rhizosphere is affected by the degree of soil salinity and the different growth stages.

LEfSe analysis was used to search for robust differential species, namely biomarkers, between soil samples from different saline-alkali sites (Figure 5). This study revealed that *o_Chitinophagales, o_Rhizobiales, o_Burkholderiales, o_Vicinamibacterales, o_Gemmatimonadales, o_Rokubacteriales and f_Nitrosomonadacea* were biomarkers of lightly saline-alkali soil. *f_Hymenobacteraceae, o_Thermoanaerobaculales, o_Longimicrobiales and g_Sphingomonas* were biomarkers of moderate saline-alkali soil. *o_Cytophagales, o_Polyangiales, o_Sphingomonadales, f_Comamonadaceae, f_Oxalobacteracea, o_Xanthomonadales, o_Actinomarinales, s_Gemmatimonadacea, o_Tistrellales, o_SBR1031, o_Gemmatimonadota, o_Kiloniellales, o_Rhodobacterales, o_Pseudomonadales, o_Gammaproteobacteri* were biomarkers of heavily saline-alkali soil. This suggests that these taxa may be important species for maintaining soil health in saline-alkali soils.

Microorganisms (including viruses, bacteria, archaea and protists) do not exist in isolation but form complex ecological interaction webs. Interactions within these ecological webs can have a positive impact (that is, a win), a negative impact (that is, a loss) or no impact on the species involved (Lidicker, 1979). Network analysis has been widely applied to explore the complex inter relationships and co-occurrence patterns of soil microbial communities (He et al., 2017; Sen and Fengzhi, 2018). Previous studies have shown that environmental changes can affect the complexity of soil microbial co-occurrence networks (Banerjee et al., 2019). In this study, we examined the effects of salinization on the co-occurrence network of bacterial communities in cotton rhizosphere soil (Figure 6). The results showed that soil salinization reduced the total nodes, average path length, graph diameter, betweenness centralization of the network. The results demonstrate that soil salinization significantly affects the diversity and structure of bacterial communities in cotton rhizosphere soil. As the degree of soil salinization increases, the relative abundance of certain beneficial bacterial groups in cotton rhizosphere soil decreases.

In this study, we combined soil nutrient analysis to compare and explain the bacterial community structure and functions in different saline-alkali soils and at different stages of cotton growth. This research provides a theoretical foundation for understanding how soil microorganisms maintain nutrient balance and drive ecosystem nutrient cycling. The findings deepen our understanding of the impact of saline-alkali soil on bacterial communities in cotton rhizosphere, and offer valuable insights for utilizing beneficial microorganisms to improve saline-alkali soil.

## Data availability statement

The original contributions presented in the study are included in the article/Supplementary material, further inquiries can be directed to the corresponding author.

## Author contributions

GF: Conceptualization, Data curation, Investigation, Writing – original draft, Writing – review & editing. YWu: Data curation, Visualization, Formal Analysis, Writing – original draft, Writing – review & editing. CY: Visualization, Validation, Writing – original draft, Writing – review & editing. QZ: Methodology, Writing – review & editing. SW: Data curation, Project administration, Writing – original draft. MD: Investigation, Writing – review & editing. YWa: Data curation, Writing – review & editing. HQ: Investigation, Writing – original draft, Writing – review & editing. LG: Data curation, Investigation, Visualization, Writing – original draft, Writing – review & editing.
