# Supplementary material for: Effects of coastal saline-alkali soil on rhizosphere microbial community and crop yield of cotton at different growth stages
Source: Front Microbiol. 2024 Apr 19;15:1359698. doi: 10.3389/fmicb.2024.1359698 (PMC11066693; doi:10.3389/fmicb.2024.1359698)
Supplement: Supplementary file 1 [file Table_1.pdf]

Supplementary Table 1 Topological properties of the co-occurrence networks

	nodes num	Average degree	Average path length	diameter	density	Clustering coefficient	Betweenness centralization	Degree centralization	modularity
**SSA**	54	3.703704	5.3219	33.3	0.06988	0.47561	0.40207	0.118798	0.59645
**MSA**	59	3.389831	3.72553	24.81818	0.058445	0.45205	0.15331	0.11397	0.6904
**HSA**	46	4.347826	2.39013	11.7889	0.096618	0.5077	0.10011	0.12560	0.63685

Note：SSA: slight saline-alkali land, MSA: moderate saline-alkali land, HSA: heavily saline-alkali land
