# Supplementary material for: Effects of coastal saline-alkali soil on rhizosphere microbial community and crop yield of cotton at different growth stages
Source: Front Microbiol. 2024 Apr 19;15:1359698. doi: 10.3389/fmicb.2024.1359698 (PMC11066693; doi:10.3389/fmicb.2024.1359698)
Supplement: Supplementary file 2 [file Table_2.pdf]

Supplementary Table 2 Relative abundance of the top 12 bacterial phyla

	SS	MS	HS	SF	MF	HF	SB	MB	HB
**Proteobacteria**	0.360896636	0.289097898	0.379133638	0.350168614	0.352078225	0.316644612	0.364381251	0.340028148	0.402857143
**Acidobacteriota**	0.225555888	0.218281037	0.136549871	0.223705477	0.197995584	0.082578181	0.252630937	0.198015853	0.101997579
**Gemmatimonadota**	0.080334409	0.129181147	0.07850265	0.093215742	0.103557442	0.099068692	0.110246922	0.127676742	0.102409201
**Bacteroidota**	0.085096952	0.092814252	0.133376696	0.035752834	0.052233161	0.078076582	0.038943964	0.065245795	0.105078692
**unclassified_Bacteria**	0.054929026	0.054704693	0.037512766	0.083484797	0.081750418	0.123825397	0.05415541	0.044721243	0.087118644
**Actinobacteriota**	0.037425134	0.05689547	0.06808953	0.044969269	0.042619771	0.084688699	0.039459888	0.062339258	0.048510896
**Chloroflexi**	0.044285464	0.04866512	0.033999173	0.039717914	0.050885091	0.098101372	0.027765604	0.052203054	0.065738499
**Myxococcota**	0.041960889	0.03530636	0.069676117	0.040774896	0.032341495	0.021557428	0.037745688	0.028015532	0.031822034
**Methylomirabilota**	0.019354273	0.029047709	0.017355201	0.024595802	0.02156303	0.004928728	0.031188457	0.016866166	0.007318402
**Nitrospirota**	0.016199862	0.013284074	0.008911637	0.018958565	0.02109334	0.008634696	0.01627658	0.010484621	0.007384988
**Others**	0.033848073	0.032582826	0.036740748	0.044588979	0.043845844	0.081820238	0.027144276	0.054000156	0.039733656
**Unknown**	0.000113394	0.000139413	0.000151972	6.71E-05	3.66E-05	7.54E-05	6.10E-05	0.000403431	3.03E-05
