# Supplementary figures and images for: Effects of coastal saline-alkali soil on rhizosphere microbial community and crop yield of cotton at different growth stages
Source: Front Microbiol. 2024 Apr 19;15:1359698. doi: 10.3389/fmicb.2024.1359698 (PMC11066693; doi:10.3389/fmicb.2024.1359698)

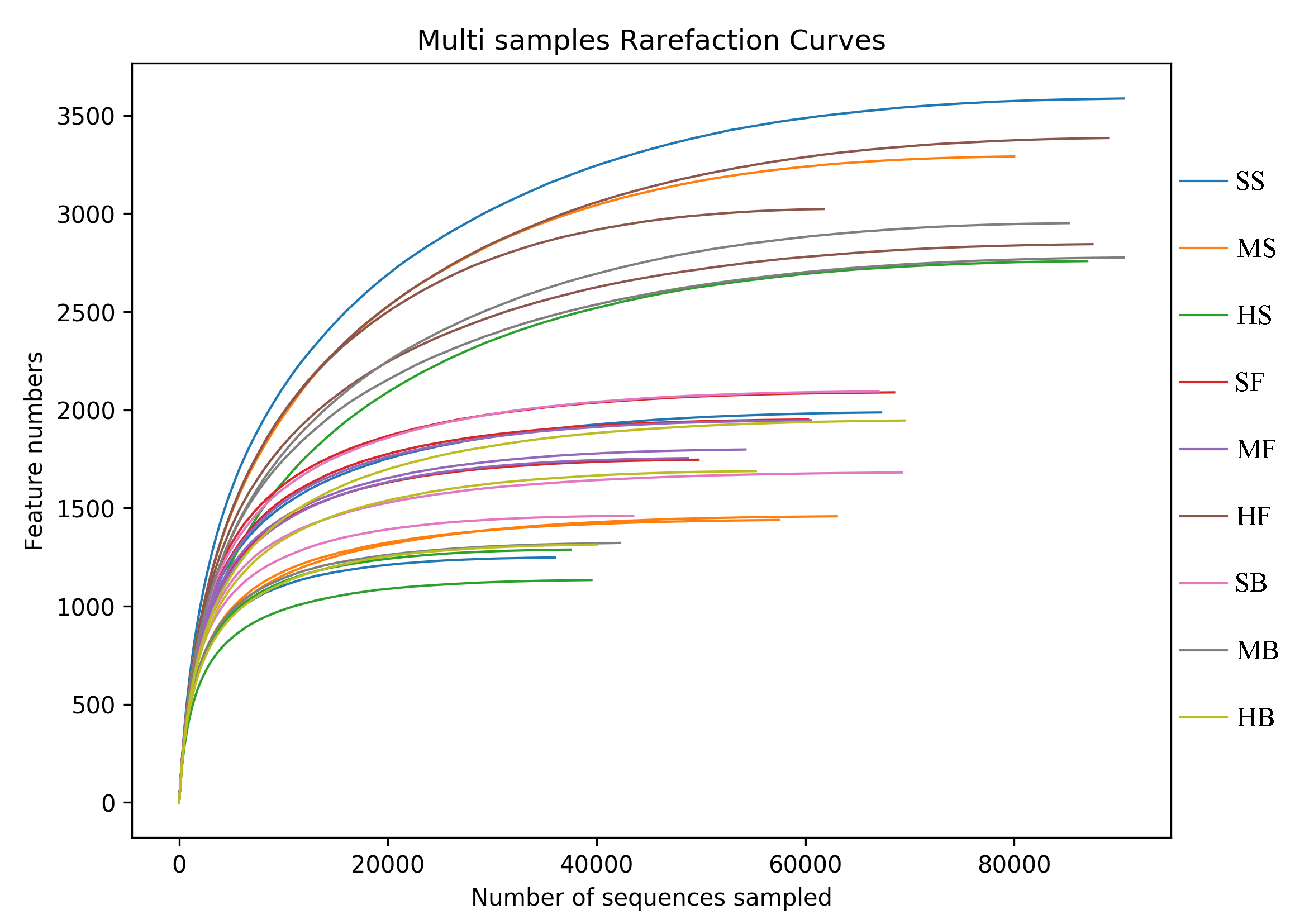

Supplement: Supplementary file 3 [file Image_1.TIF]

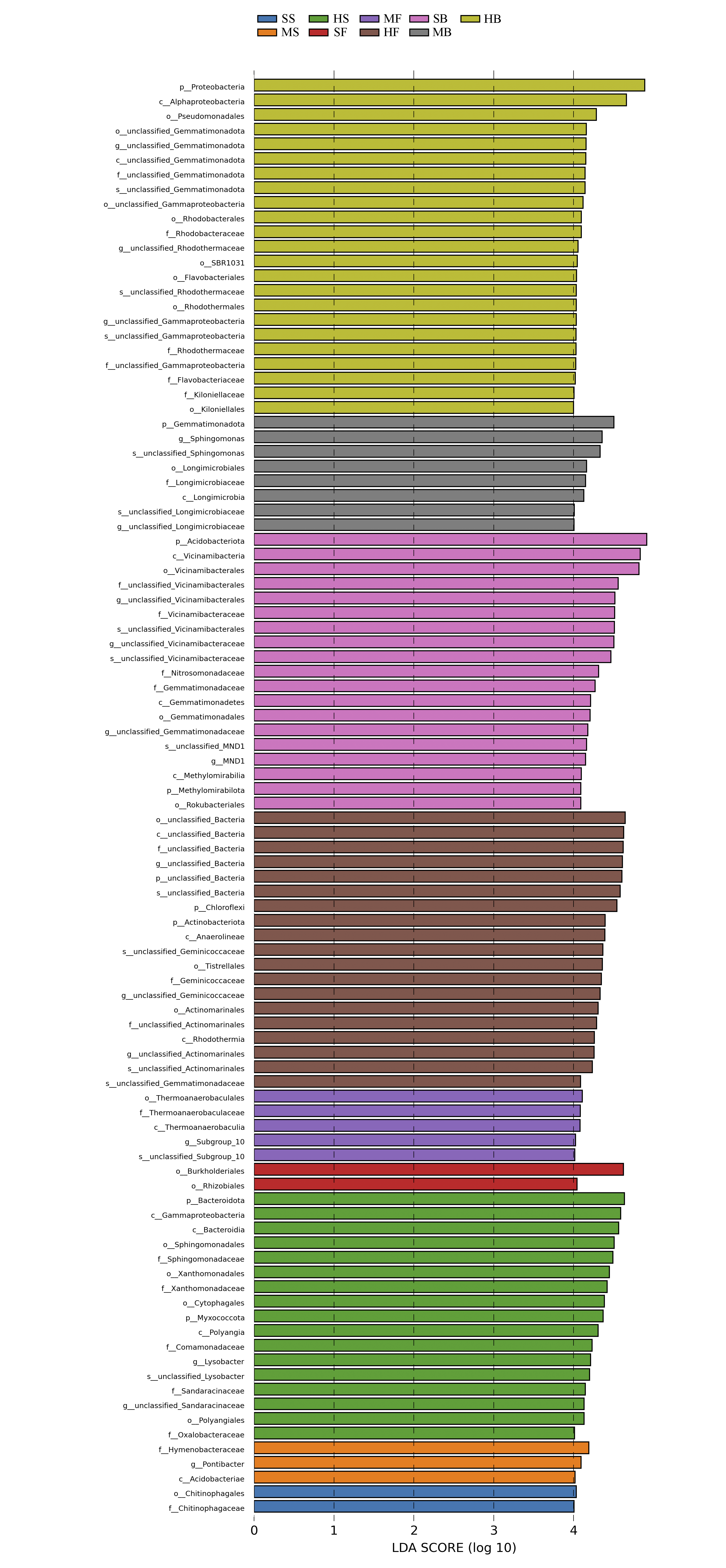

Supplement: Supplementary file 4 [file Image_2.TIF]
